# 原发性肺淋巴上皮瘤样癌8例临床分析

**DOI:** 10.3779/j.issn.1009-3419.2020.03.06

**Published:** 2020-03-20

**Authors:** 惠 赵, 建华 陈

**Affiliations:** 410006 长沙，中南大学湘雅医学院附属肿瘤医院/湖南省肿瘤医院胸内一科 Department of Thoracic Medicine, Affiliated Cancer Hospital of Xiangya School of Medicine, Central South University, Hunan Cancer Hospital, Changsha 410006, China

**Keywords:** 淋巴上皮瘤样癌, 肺肿瘤, 临床病理, 预后, Lymphoepithelioma-like carcinoma, Lung neoplasms, Clinicopathological features, Prognosis

## Abstract

**背景与目的:**

淋巴上皮瘤样癌为罕见的上皮肿瘤，多来源于鼻咽部，也发生在前肠起源器官，如肺、胃、唾液腺和胸腺。原发性肺淋巴上皮瘤样癌（primary pulmonary lymphoepithelioma-like carcinoma, PPLELC）是一种罕见的非小细胞肺癌（non-small cell lung cancer, NSCLC），约占肺癌的0.9%。本文目的在于探讨PPLELC的临床病理特点、治疗方法以及预后情况。

**方法:**

回顾性分析湖南省肿瘤医院2013年10月-2016年6月收治的PPLELC患者8例，对其临床资料及预后进行临床分析，以深入研究这种罕见的肺癌亚型。

**结果:**

8例患者中，62.5%（5/8）为女性，中位年龄为51.5岁（41岁-64岁），大多数（87.5%）患者不吸烟，50.0%患者无症状，约37.5%为Ⅰ期，50.0%为Ⅲ期，12.5%为Ⅳ期。PPLELC的典型病理特征与未分化的鼻咽癌相同，其特点是分化较差的肿瘤细胞，具有大的泡状核和核仁突出，呈合胞体生长，伴有淋巴细胞浸润。免疫表型：癌细胞P63（100.0%, 6/6）、CK5/6（100.0%, 5/5）、CK（100.0%, 5/5）阳性。对8例患者的程序性死亡配体-1（programmed cell death-ligand 1, PD-L1）表达进行了检测，当≥5%的细胞膜染色时定义为PD-L1阳性，PD-L1表达频率为50.0%（4/8），肿瘤细胞阳性比例分数（tumor proportion score, TPS）波动在20.0%-70.0%。3例行表皮生长因子受体（epidermal growth factor receptor, *EGFR*）基因突变检测，结果呈阴性；2例行*EML4-ALK*融合基因检测，结果呈阴性；1例行KRAS、B-raf、C-kit、HER2、VEGFR1、VEGFR2检测，结果示KRAS野生型，B-raf、C-kit无突变，HER2、VEGFR1、VEGFR2中等表达。所有患者均接受了手术治疗，其中接受了辅助化疗者5例，其中Ib期2例，Ⅲa期2例，Ⅳ期1例；辅助放化疗者1例，分期为Ⅲa期，接受了新辅助化疗者1例，分期为Ⅲa期。截止至随访截止期，所有患者均存活，中位存活时间为57个月，3年和5年总生存率（overall survival, OS）分别为87.5%和50.0%；无病生存率（disease-free survival, DFS）分别为87.5%和50.0%。

**结论:**

PPLELC是一种罕见但独特的NSCLC亚型，具有明显的临床病理学特征，通常发生于年轻的非吸烟患者，无性别倾向，并与EB病毒（Epstein-Barr virus, EBV）感染有关，组织形态及免疫组化是诊断的主要手段。大多数患者缺乏EGFR和ALK等常见基因突变，提示这些基因的突变与PPLELC的发生无关。PD-1和PD-L1可能是潜在的治疗靶点。与其他类型的NSCLC相比，大多数患者处于早期或局部晚期，预后较好。目前还没有针对这种罕见肿瘤的标准化治疗方案。早期以手术切除为主，中晚期或转移阶段则采用手术、化疗、放疗等多种治疗方式。由于其发病率低，为了确定其生物学特性和最佳治疗方案，还需要进一步的研究。

## Abstract (en)

**Background and objective:**

Lymphoepithelioma-like carcinoma, an uncommon epithelial tumor, is mostly originated form the nasopharynx and also occurs in foregut-derived organs, such as lung, stomach, salivary gland, and thymus. Primary pulmonary lymphoepithelioma-like carcinoma (PPLELC) is a rare subtype accounting for around 0.9% of non-small cell lung cancer (NSCLC). We aimed to evaluate clinicopathological features, treatment modalities, and prognosis of PPLELC.

**Methods:**

In the current study, a retrospective analysis on 8 patients diagnosed with PPLELC at Hunan Cancer Hospital between October 2013 and June 2016 was conducted with respect to their clinical characteristics and outcomes, in order to deeply investigate this rare subtype of lung cancer.

**Results:**

In all 8 patients, 62.5% (5/8) were female, and the median age was 51.5 years (range 41 years-64 years). The majority (87.5%) were never smokers and 50.0% were asymptomatic at diagnosis. About 37.5% presented with stage I disease, 50.0% had stage Ⅲ disease and 12.5% had stage Ⅳ disease. Histologically, the tumor morphology was indistinguishable from undifferentiated carcinoma of the nasopharynx and were characterized by poorly differentiated tumor cells with large vesicular nuclei and prominent nucleoli showing syncytial growth patterns and accompanied by heavy lymphocytic infiltration. The tumor cells were presented as positive for P63 (100.0%, 6/6), CK5/6 (100.0%, 5/5), CK (100.0%, 5/5). The expression of programmed cell death ligand 1 (PD-L1) in 8 patients was detected. When membranous staining was present in ≥5% of the cells, it was defined as PD-L1 positive. The PD-L1 expression frequency was 50.0% (4/8), and the tumor proportion score (TPS) fluctuated between 20.0% and 70.0%. Epidermal growth factor receptor(*EGFR*) mutations were detected in 3 cases and all tested samples were wild type;  moreover, *ALK* rearrangement was negative in 2 patients with available data. KRAS, *B-raf*, *C-kit*, HER2, VEGFR1 and VEGFR2 were detected in 1 case. The results showed that KRAS was wild type, *B-raf* and *C-kit* mutation was negative, and HER2, VEGFR1 and VEGFR2 were moderately expressed. All patients underwent surgery with or without adjuvant therapy. Among these patients, 5 patients received adjuvant chemotherapy, including 2 patients with stage Ib disease, 2 patients with stage Ⅲa disease and 1 patient with stage Ⅳ disease. 1 patient with stage Ⅲa disease received adjuvant chemotherapy and radiotherapy and 1 patient with stage Ⅲa disease received neoadjuvant chemotherapy. All patients survived until the deadline for follow-up. The median survival time of all LELC patients is 57 months. The 3- and 5-year overall survival (OS) rates of LELC patients were 87.5% and 50.0%, disease-free survival (DFS) rates were 87.5% and 50.0%, respectively.

**Conclusion:**

PPLELC is uncommon but distinct subtype of NSCLC with unique clinicopathologic characteristics that tends to affect young nonsmoking patients, without significant predilection for sex and with strong association with Epstein-Barr virus (EBV) infection. Histology and immunohistochemistry are the main diagnostic methods. Rare or no driver gene mutations were found in the common oncogenes such as *EGFR* mutations and *ALK* gene rearrangement, implying that the mutagenesis of these genes was not involved in the tumorigenesis of PPLELC. PD-1 and PD-L1 may be potential therapeutic targets for PPLELC. The patients are diagnosed at an earlier stage and have a better prognosis than those with other non-small cell lung cancer. No standardized treatment regimens currently exist for this rare tumor. The mainstay of treatment for early-stage disease is curative surgical resection, whereas multimodality treatment (surgery, chemotherapy, radiotherapy) has been adopted in advanced or metastatic diseases. Due to its low incidence, further research is needed to determine its biological characteristics and optimal treatment options.

淋巴上皮瘤样癌在鼻咽、胃、胸腺、肝、宫颈、唾液腺和膀胱中均有报道^[1]^。原发性肺淋巴上皮瘤样癌（primary pulmonary lymphoepithelioma-like carcinoma, PPLELC）是一种罕见的原发性肺癌亚型，组织学上类似于未分化的鼻咽癌（nasopharyngeal carcinoma, NPC）^[2]^。于1987年由Begin等^[3]^首次描述，PPLELC已被认为与Epstein-Barr病毒（Epstein-Barr virus, EBV）感染密切相关^[4]^。在2015年世界卫生组织（World Health Organization, WHO）肺部肿瘤分类中，PPLELC被归类为其他和未分类癌^[5]^。PPLELC通常影响从不吸烟者，无性别倾向，比非小细胞肺癌（non-small cell lung cancer, NSCLC）更年轻^[6]^。文献报道了多种治疗方法，但由于其罕见性，对PPLELC的治疗均是经验性的，且仍有争议。本文总结了8例PPLELC患者的临床病例特点、治疗方法以及预后情况，并进行相关文献的复习，现报道如下。

## 1 对象与方法

本回顾性研究收集了2013年10月-2016年6月湖南省肿瘤医院收治的PPLELC患者8例，所有患者均具有完整的临床资料，手术或活检组织经苏木精-伊红（hematoxylin-eosin, HE）常规染色结合多项免疫组化指标等进行病理诊断。所有病例均根据PPLELC的诊断标准^[7]^进行诊断。术后分期使用最新第8版肺癌肿瘤原发灶-淋巴结-转移（tumor-node-metastasis, TNM）分期系统进行分期^[8]^。所有患者均接受鼻咽内镜检查，以排除鼻咽部转移性淋巴上皮瘤样癌。收集患者的性别、年龄、吸烟史、饮酒史、症状、肿瘤标志物、血清病毒学检测、支气管镜检查、影像学表现、肿瘤部位、肿瘤大小、转移部位、分期、治疗方法及预后等资料进行分析。患者的随访信息通过住院病历资料、门诊资料及电话随访获得，随访生存期自明确诊断第1天计算，随访截止日期为2019年8月20日。

## 2 结果

### 2.1 临床特征

在8例患者中，3例为男性，5例为女性，患者年龄介于41岁-64岁之间，中位年龄为51.5岁，所有患者均来自于湖南地区。1例（12.5%）患者既往有吸烟史，1例（12.5%）患者有饮酒史，所有患者均无家族史。体能状态（performance status, PS）评分0分者5例，1分者3例。其中4例患者无任何临床症状，均为体检中发现肺部肿块，3例表现为咳嗽、咳痰，1例表现为胸痛、胸闷、气促。肿瘤位于左上肺1例（12.5%），左下肺2例（25.0%），右上肺1例（12.5%），右中肺4例（50.0%），其中7例为单发病灶，另外1例患者伴有左下肺转移灶。根据最新第8版肺癌TNM分期，术后分期如下：Ia期1例、Ib期2例、Ⅲa期4例、Ⅳ期1例。8例患者术前均完善纤维支气管镜检查，其中5例镜下未见明显异常，3例镜下均见支气管管壁粘膜肿胀、充血，管腔狭窄。8例患者中，所有患者均完善了鼻咽镜检查，均未见明显异常。8例患者中，共有3例患者进行了基因检测，1例患者在初治时进行了表皮生长因子受体（epidermal growth factor receptor, EGFR）检测，结果为EGFR野生型，1例患者在复发后完善基因检测结果为EGFR野生型、ALK阴性，另有1例患者在复发后完善基因检测的结果为*EGFR*、*KRAS*野生型，*ALK*阴性，*B-raf*、*C-kit*无突变，*HER2*、*VEGFR1*、*VEGFR2*中等表达。所有患者在术前均进行了肺功能检查，其中3例提示轻度限制性通气功能障碍，1例提示中重度限制性通气功能障碍，余正常。

**1:** 8例患者临床资料 Clinical features of 8 patients

Case	Gender	Age (yr)	EBV serology	Tumor location	Tumor size (cm)	Invaded sites	Lymph node metastasis (group)	Stage	Treatment	OS (mo)
1	F	50	-	LLL	1.5×1.5×1.8	No	No	Ⅰa	S	69
2	F	64	ND	RML	5.0×4.5×4.0	No	No	Ⅰb	S+CT	63
3	M	41	ND	RML	2.0×1.8×1.7	No	No	Ⅰb	S+CT	74
4	M	54	ND	LLL	4.5×4.0×4.0	Bronchia	4, 5, 10	Ⅲa	S+CT	35
5	F	53	+	RML	3.3×3.0×2.5	No	4	Ⅲa	S+CT+RT	40
6	M	43	+	RUL	2.0×1.5×1.5	No	10, 11, 12	Ⅲa	NCT+S	41
7	F	46	+	RML	5.0×4.5×4.0	No	7, 10	Ⅲa	S+CT	51
8	F	55	-	LUL+LLL	5.0×4.5×2.0	Pericardial tissue	4, 11	Ⅳ	S+CT	70
F: female; M: male; +: positive; -: negative; ND: not done; RUL: right upper lobe of lung; RML: right middle lobe of lung; RLL: right lower lobe of lung; LUL: left upper lobe of lung; LLL: left lower lobe of lung; Station 4: lower paratracheal nodes; Station 5: subaortic nodes (aorto-pulmonary window); Station 7: subcarinal nodes; Station 10: hilar nodes; Station 11: interlobar nodes; Station 12: lobar nodes; S: surgery; NCT: neoadjuvant chemotherapy; CT: chemotherapy; RT: radiotherapy; EBV: Epstein-Barr virus; OS: overall survival.										

### 2.2 实验室指标

所有患者在首诊时均接受了肿瘤标志物检查，糖类抗原125（carbohydrate antigen 125, CA125）升高者1例，癌胚抗原（carcinoembryonic antigen, CEA）升高者1例，细胞角蛋白19片段（cytokeratin 19 fragment, CYFRA21-1）升高者1例，其余5例肿瘤标志物均正常。在PPLELC诊断时，所有患者的乳酸脱氢酶（lactate dehydrogenase, LDH）及白蛋白（albumin, ALB）水平均正常。在8例患者中，有5例患者进行了EBV的血清检测，结果提示EBV衣壳抗原抗体IgA阳性者3例，EBV感染阳性率为60.0%。

### 2.3 影像学表现

所有患者在诊断时均接受了肺部计算机断层扫描（computed tomography, CT）检查，其中左上肺1例，左下肺2例，右上肺1例，右中肺4例，周围型5例，中央型3例，4例边界清晰，3例呈分叶状，3例密度不均匀，肿块最大直径为1.3 cm-5.9 cm，平均最大径为（3.54±1.39）cm。1例患者伴有阻塞性肺炎，1例患者伴有同侧少许胸腔积液，1例患者转移了至同侧不同肺叶，3例伴有纵隔、肺门淋巴结转移。所有患者肿块内均未见空气支气管征、血管包埋征、空洞、钙化等征象。共有2例患者在初治时接受了胸部正电子发射计算机断层显像（positron emission tomography-computed tomography, PET-CT）检查，1例于右中肺见一大小约3.3 cm×3.1 cm×2.8 cm团块状异常放射性浓聚影，边缘呈分叶状，最大标准摄取值（standard uptake value, SUV）为6.5；1例于右中肺近肺门区见一团块状异常放射性浓聚影，大小约3.7 cm×2.7 cm，最大SUV为6.2，右中肺见斑片状影，最大SUV为1.1，奇静脉食管隐窝、右侧肺门见多个肿大淋巴结，最大SUV为6.4。此外，还有1例患者在复发时接受了胸部PET-CT检查，提示：右侧胸膜见多个软组织密度结节影，PET-CT见异常放射性浓聚影，最大SUV为3.6，考虑右胸膜转移性病变。

### 2.4 病理结果

8例患者均进行了手术治疗，均行术后常规病理检测，8例患者病理均证实为PPLELC。对8例患者的手术病理情况进行复习，结果显示肿瘤侵犯支气管1例，累及心包组织1例，淋巴结转移者5例，其中，4组淋巴结受侵3例，5组淋巴结受侵1例，7组淋巴结受侵1例，10组淋巴结受侵3例，11组淋巴结受侵2例，12组淋巴结受侵1例。6例患者进行了肺癌相关免疫组化标志物检测，检测的指标及其阳性数包括CK（5/5），鳞癌标记物P63（6/6）、CK5/6（5/5）、P40（1/1），腺癌标记物TTF-1（0/5）、CK7（0/4）、EMA（0/1）、Napsin A（0/1），神经内分泌标记物SYN（0/6）、CGA（0/5）、S100（1/2）、CD56（1/3），淋巴组织源性标记物CD45（0/2）、CD3（0/1）、CD20（0/1）、CD5（0/1），间叶组织标记物Vimentin（1/3），肌上皮标记物Calponin（0/1），上皮性肿瘤标记物HMB45（0/1），Ki-67约5%-40%。我们使用PD-L1抗体对8例患者的石蜡包埋的肿瘤切片进行染色，当≥5%的细胞膜染色时定义为PD-L1阳性。8例患者中有4例（50.0%）检测到PD-L1阳性，肿瘤细胞阳性比例分数（tumor proportion score,  TPS）波动在20.0%-70.0%。病理特点：肉眼观察，PPLELC多为单发，表现为圆形或卵圆形结节，肿块最大直径为1.5 cm-4.5 cm，无包膜，境界欠清，质地中等或硬，切面呈灰黄色或灰白色，其中1例伴部分坏死，未伴发出血。镜下见肿瘤细胞呈大小不等的片块状或巢状排列，癌细胞呈圆形或卵圆形，呈合体细胞样，胞质中等量，淡染或弱嗜酸性，核大呈空泡状，染色深，外形不规则，可见1个-2个明显嗜酸性核仁，间质淋巴细胞或浆细胞浸润明显。

### 2.5 治疗情况

所有患者均进行了肺癌根治术+淋巴结清扫术，其中1例拟行右上肺癌根治术的患者因术中见肿瘤侵犯右上叶支气管及右中间支气管，并侵犯右上肺动脉干，无法分离出右上肺后段动脉，改行右全肺切除术；1例合并左下肺转移灶及心包组织受累的患者同时进行了左下肺部分切除+心包部分切除。术中发现胸腔粘连者5例，肿块侵犯脏层胸膜者1例，心包组织受累者1例，同侧不同肺叶（左下肺）转移灶者1例。在手术方式上，2例患者行电视辅助胸腔镜手术（video-assisted thoracic surgery,  VATS），6例行开胸手术。所有患者手术切缘均为阴性。8例患者中，1例进行了新辅助化疗，分期为Ⅲa期，方案为吉西他滨+顺铂，2个周期；5例进行了术后辅助化疗，其中Ib期2例，Ⅲa期2例，Ⅳ期1例，均为含铂双联方案，联合吉西他滨2例，联合紫杉醇2例，联合培美曲塞1例；1例Ⅲa期患者进行了术后辅助放化疗，化疗方案为多西他赛+顺铂，疗程为6个周期。2例患者复发，1例患者在术后49个月（2019年6月）于外院复查发现了右纵隔淋巴结转移复发，复发分期是rT0N2M0，1例患者在术后17个月（2014年10月）发现了右胸膜转移，随后均进行积极的同步放射治疗+化疗，化疗方案分别为紫杉醇+铂类、长春瑞滨+铂类，目前维持治疗中，2例患者随访至截止日期均仍存活。

**1:**
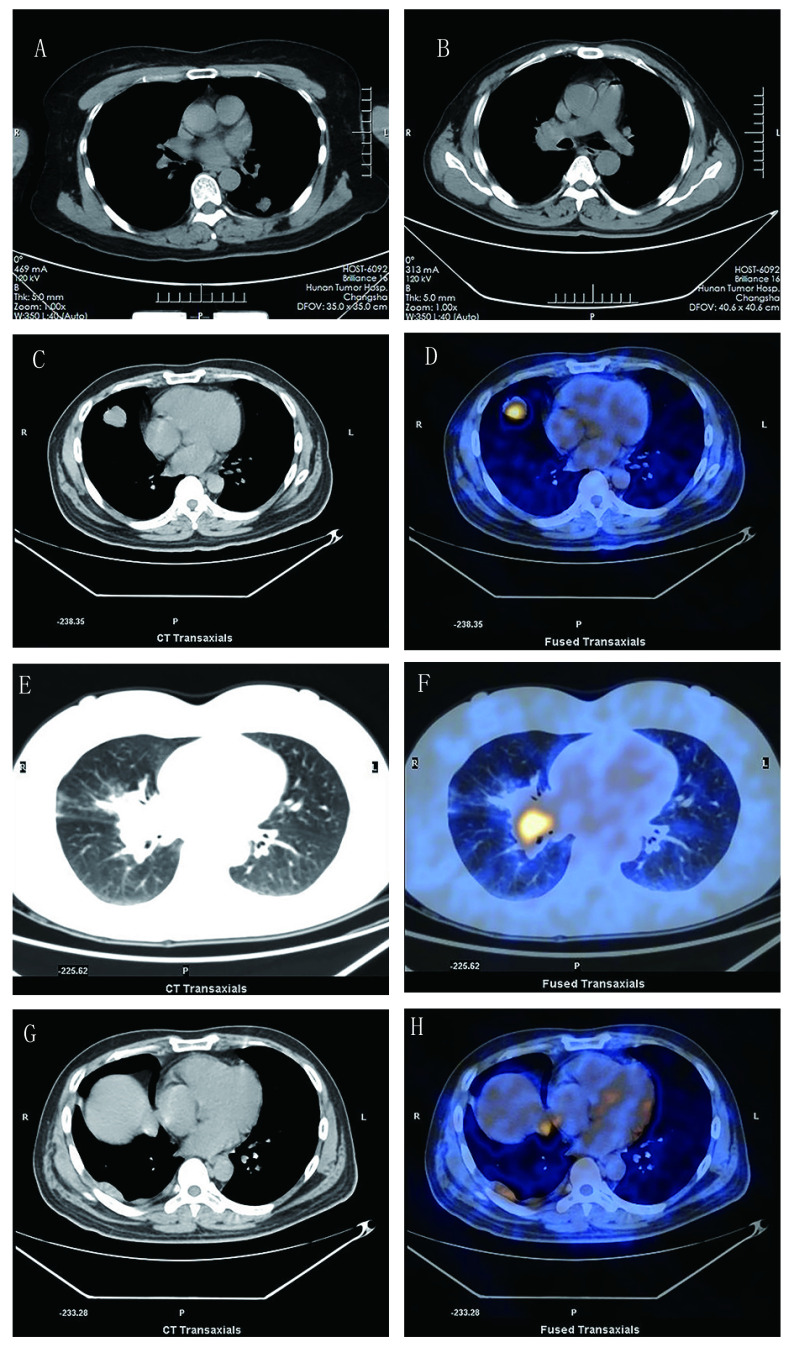
PPLELC的影像学表现。A：左肺下叶软组织结节影，分叶状，边界较清晰，密度欠均，左侧少量胸腔积液；B：化疗后CT片，右肺门区类圆形肿块影，边界不清，密度欠均；C、D：右中肺分叶状团块状影，相对应的PET-CT；E、F：右中肺近肺门区肿块影，右中肺斑片状影，相对应的PET-CT；G、H：右侧胸膜多个软组织密度结节影，相对应的PET-CT Imaging findings of PPLELC. A: A soft tissue nodule in the left lower lobe with lobular, clear boundary, inhomogeneous density and a limited amount of effusion in the left side of the chest; B: A round tumor in the right hilar area with unclear boundary and nonuniform density was demonstrated in the CT film after chemotherapy; C and D: An lobulated mass lesion in the right middle lobe of lung and the corresponding PET-CT; E and F: Mass shadow near hilum of right middle lung, patchy shadow of right middle lung and the corresponding PET-CT; G and H: Multiple soft tissue nodules were observed in the right side of the pleura and the corresponding PET-CT. PET-CT: positron emission tomography-computed tomography; PPLELC: primary pulmonary lymphoepithelioma-like carcinoma

**2:**
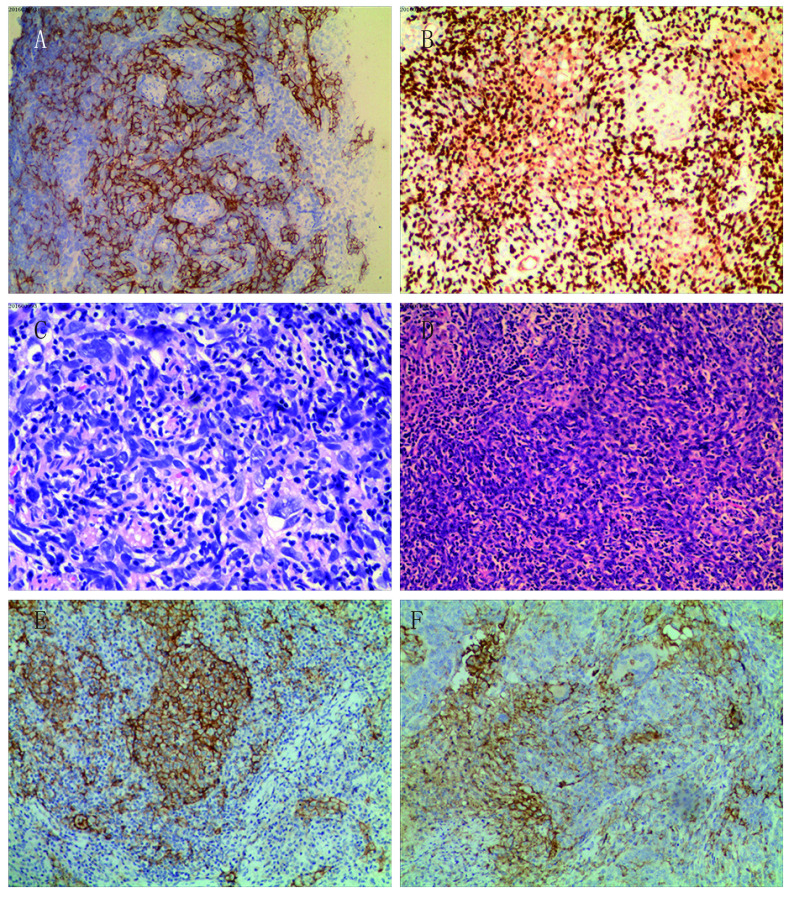
PPLELC的免疫组织化学检查。A：免疫组织化学染色癌细胞CK5/6表达阳性（×200）；B：免疫组织化学染色癌细胞P63表达阳性（×200）；C：癌细胞体积大，胞质淡染或弱嗜酸性，核呈空泡状，核仁清晰（×400）；D：癌细胞呈巢状分布，间质有丰富的淋巴细胞浸润（×200）；E：PD-L1免疫组化染色呈膜阳性（TPS: 70%）（×200）；F：PD-L1免疫组化染色呈膜阳性（TPS: 30%）（×200） Immunohistochemistry of PPLELC. A: Immunohistochemistry showing tumor cells positive for CK5/6 (×200); B: Immuno-histochemistry showing tumor cells positive for P63 (×200); C: The tumor cells are bulky, with light stained or weakly eosinophilic cytoplasm, vacuolated nuclei, and clear nucleoli; D: The distribution of carcinoma cells showed as nests with abundant lymphocyte infiltration in the stroma; E: Positive PD-L1 immunohistochemical staining with a membranous pattern (TPS: 70%) (×200); F: Positive PD-L1 immunohistochemical staining with a membranous pattern (TPS: 30%) (×200). PD-L1: programmed cell death ligand; TPS: tumor proportion score

### 2.6 随访结果

随访截止日期至2019年8月20日，所有患者均获得了完整的随访数据。随访时间1个月-34个月，平均随访时间为7.75个月，所有患者均存活。总生存期（overall survival, OS）定义为从确诊起至因任何原因死亡或最后一次随访时间，无病生存期（disease-free survival, DFS）定义为从确诊起至疾病复发的时间。最长存活时间为74个月，最短存活时间为35个月，中位存活时间为57个月。3年和5年OS率分别为87.5%和50.0%；DFS率分别为87.5%和50.0%。

## 3 讨论

PPLELC是一种典型的罕见肺癌，其发生率约占其他肺恶性肿瘤的0.92%^[9]^。据报道，PPLELC常见于相对年轻的不吸烟的亚洲人群，无明显的性别差异^[7]^。PPLELC的致癌机制仍存在争议，有研究^[10]^表明在西方人群中，EBV感染与PPLELC之间没有明显的相关性，然而免疫组化和分子生物学研究^[11]^表明，EBV与B淋巴细胞的CD受体相互作用导致上皮细胞增殖，从而导致EBV诱导的增生和癌前病变。而且，Chang等^[9]^发现EBV血清学效价越高，与肿瘤分期越高、肿瘤体积越大有关。Ngan等^[12]^发现循环EBV DNA水平与临床治疗反应及肿瘤复发密切相关。Xie等^[13]^报道高浓度EBV DNA是肺LELC患者独立的不良预后指标。在我们的研究中，有5例患者进行了EBV的血清检测，EBV阳性率为60.0%。女性患者（5/8, 62.5%）居多，这与Chang等^[14]^的报告一致，但与其他公布的男性/女性比例2.2:1^[4]^和6:5^[15]^不同。患者的年龄范围为41岁-64岁（平均50岁），与之前的报告^[4]^大致相似。PPLELC患者的平均年龄比其他组织学类型的NSCLC年轻10岁^[9]^。最常见的症状是单纯咳嗽或是伴有其他症状的咳嗽^[7]^，约20%-35%的病例在诊断时无症状，在常规胸片检查中偶然发现肿瘤^[7, 16, 17]^。在我们的8例患者中，近50.0%的患者无症状，最常见的症状为咳嗽（37.5%）。非吸烟者7例（87.5%），吸烟者1例（12.5%），Ho等^[7]^报道的非吸烟率为69%，PPLELC患者的吸烟率较其他肺癌患者低（> 60%），这表明除吸烟外可能还有另外的发病机制^[14]^。

监测血清肿瘤标志物可能对肺癌的诊断、治疗和疾病监测具有重要意义。Xia等^[18]^报道，77.8%（7/9）的PPLELC患者CA-125升高，提示CA-125升高可能是PPLELC的指标之一。Liang等^[17]^报道，CYFRA21-1阳性的患者占62.5%，NSE阳性的患者占55%，血清CYFRA21-1和NSE水平可能与疾病进展有关，可用于检测早期肿瘤复发，建议治疗期间定期随访。在我们的报道中，阳性率最高的是CA125（25.0%, 2/8），CEA阳性率为12.5%（1/8），CYFRA21-1阳性率为12.5%（1/8）。Liang等^[17]^还发现早期PPLELC患者血清LDH和血清ALB水平正常，无淋巴结转移，行完整切除者OS明显改善（*P* < 0.05）；血清ALB水平是*Cox*回归模型中独立的预后因子（*P*=0.005）。在我们的报道中，所有患者的LDH及ALB血清水平均正常。

影像学对于PPLELC的诊断及鉴别诊断也有一定的意义。根据Ooi等^[19]^的报道，晚期PPLELC具有其放射学特征，肿瘤可表现为直径大、边界清晰，与纵隔密切相关，且伴有支气管血管周围淋巴结转移和血管包绕。Hoxworth等^[20]^认为PPLELC主要表现为胸膜附近的孤立性肺结节，体积 < 3.5 cm，通常不累及淋巴结。Ma等^[21]^报道PPLELC的CT表现包括界限明确（63.4%）、分叶（78.0%）、血管或支气管包裹（43.9%）、阻塞性肺炎（41.5%）、胸腔积液（12.2%）和钙化（4.9%），在右中叶（31.7%）和左下叶（29.3%）更常发生。在我们报道的病例中，右中肺最多见（4/8, 50.0%），此外可见到的特征有边界清晰（4/8, 50.0%）、分叶状（4/8, 50.0%）、密度不均匀（3/8, 37.5%）、阻塞性肺炎（1/8, 12.5%）、胸腔积液（1/8, 12.5%），肿瘤大小从1.3 cm-5.9 cm不等。在8例患者中，25.0%（2/8）的患者发现淋巴结受累，低于Ooi等^[19]^（100.0%）和Huang等^[22]^（81.3%）的报道。

PPLELC的诊断主要依赖于形态学特征，典型的PPLELC由未分化的癌细胞组成，细胞质边界不明确，排列在合胞体片和巢状。肿瘤细胞核呈圆形、卵圆形或细长，细胞核轮廓略不规则，呈泡状染色质，核仁明显^[4]^。间质表现为较厚的纤维带，含有大量的反应性淋巴浆细胞和其他炎症细胞^[4, 15]^。根据Wang等^[23]^的报道，PPLELC肿瘤细胞均显示CK5/6和P63阳性表达，但几乎所有患者的TTF-1阴性（34/34, 100.0%）或CK7阴性（34/35, 97.1%）。本组患者的CK、CK5/6、P63表达均呈阳性，提示PPLELC起源于上皮组织，应属于鳞状细胞癌。其他TTF-1、CK7、EMA、Napsin A、SYN、CGA、S100、CD56、CD45、CD3、CD20、CD5、Vimentin、Calponin、HMB45、ALK大都为阴性，部分偶见散在阳性。

据报道^[23, 24]^，PPLELC缺乏*EGFR*突变和*ALK*基因重排等常见驱动基因突变，Liang等^[17]^报道39例*EGFR*突变全为阴性，Chang等^[14]^报道只有17.4%的PPLELC患者存在*EGFR*突变，Tam等^[25]^报道了11例肺LELC患者中的1例*EGFR*突变。Wong等^[26]^报道了11例PPLELC患者中均未发现*EML4-ALK*融合基因。此外，免疫治疗开启了肿瘤治疗，尤其是肺癌治疗的新时代，但PD-L1表达对PPLELC这种罕见人群的预后意义仍存在争议。2015年3项病例报道显示大部分（63.3%-75.8%）PPLELCs患者表达PD-L1^[24, 27, 28]^。Jiang等^[28]^报道PD-L1表达阳性患者较阴性患者具有更长的PFS和OS。相比之下，Chang等^[27]^报道PD-L1高表达患者较低表达患者DFS低。Fang等^[24]^观察到PD-L1表达与患者预后无显著相关性。在我们的报道中，50.0%（4/8）的PPLELCs患者PD-L1表达阳性，稍低于以往的报道。

大多数患者在疾病早期阶段得到诊断，并且接受了原发肿瘤的手术治疗^[6]^，晚期或转移性疾病通常采用多模式治疗（外科、化疗、放疗）^[7]^。既往研究^[25, 29]^报道PPLELC的早期和晚期对化疗均有较理想的反应。辅助化疗已经被证实可以显著改善接受完整切除的Ⅲa期PPLELC患者的预后^[17]^。由于缺乏共识，化疗方案没有一致性，基于铂类药物的方案是首选。最常用的化疗方案包括顺铂或卡铂联合5-氟尿嘧啶、紫杉醇、多西紫杉醇或吉西他滨^[30]^。在某些报告中，放射疗法也被证明是有益的^[30]^。在我们报道的患者中，均以手术治疗为主，有1例患者术前接受了新辅助化疗并获得了部分缓解，有6例患者术后接受了辅助化疗或联合放疗。有研究^[7, 15, 22]^结果表明，与其他非小细胞癌相比，PPLELC淋巴结扩散或远处转移的发生率较低，预后较好。根据Liang等^[17]^的报告，2年和5年OS率分别为88%和62%。Jiang等^[28]^报道3年和5年PFS率分别为76.0%和68.0%，3年和5年OS率分别为88.0%和79.0%。另外，He等^[31]^报道PPLELC患者的中位生存期为107个月，1年、3年和5年OS率分别为85.6%、74.5%和55.2%，与其他类型的NSCLC相比，其OS优于大细胞肺癌（large cell lung cancer, LCLC）、腺癌（adenocarcinoma, AD）和鳞状细胞肺癌（squamous cell carcinoma, SCC）。Jiang等^[32]^报道，单因素分析显示，T、N分级和TNM分期影响OS，N分级和TNM分期影响DFS；多因素分析未发现影响OS或DFS的独立因素。经Cox回归分析，吸烟^[24]^和肿瘤分期^[24, 28]^是影响OS的两个独立因素；而肿瘤的直径和分期^[28]^是影响DFS的独立因素。PPLELC细胞附近存在大量CD8阳性细胞毒性T淋巴细胞，以及肿瘤细胞中p53和cerb B-2癌蛋白低表达，已被认为是PPLELC预后较好的原因^[9]^。本研究预后令人满意，中位存活时间为57个月，3年和5年OS率分别为87.5%和50.0%，DFS率分别为87.5%和50.0%。

总之，PPLELC具有独特的临床和病理特征，常见于相对年轻的人群，无明显性别差异，与吸烟无关，临床症状与影像学无明显特异性，诊断主要依赖于病理学，PPLELC与未分化鼻咽癌的病理相似，其与潜在EBV感染的关系对诊断和治疗具有重要意义。与其他类型NSCLC不同，PPLELC较少发生远处转移，多在早期得到诊断，且无论分期如何，都可以从更积极的多学科治疗中受益，即手术、化疗和放疗。此外，靶向治疗可能不会为PPLELC患者提供明确的益处。PPLELC免疫治疗具有潜在的研究价值，其有可能成为晚期PPLELC的新标准治疗方法。PPLELC患者预后优于其他类型的NSCLC。为了更全面地了解PPLELC，需要积累更多病例，分享PPLELC的临床经验。

## Author contributions

Chen JH conceived and designed the study. Chen JH and Zhao H conducted the histological evaluation and immunohistochemical evaluation. Zhao H collected the data. Zhao H analyzed and interpretated the data. Zhao H drafted the manuscript. Chen JH reviewed and revised the manuscript. All the authors had access to the data. All authors read and approved the final manuscript as submitted.
